# Delayed acquisition of airway commensals in antibiotic naïve children and its relationship with wheezing in rural Ecuador

**DOI:** 10.3389/falgy.2023.1214951

**Published:** 2023-08-10

**Authors:** Paul A. Cardenas, Michael J. Cox, Saffron A. Willis-Owen, Miriam F. Moffatt, William O. Cookson, Philip J. Cooper

**Affiliations:** ^1^ National Heart and Lung Institute, Imperial College London, London, United Kingdom; ^2^ Instituto de Microbiología, COCIBA, Universidad San Francisco de Quito, Quito, Ecuador; ^3^ Institute of Infection and Immunity, St George’s University of London, London, United Kingdom; ^4^ Escuela de Medicina, Universidad Internacional del Ecuador, Quito, Ecuador

**Keywords:** asthma, wheeze, Ecuador, rural, microbiome, microbiota, hygiene hypothesis

## Abstract

**Introduction:**

The hygiene hypothesis identified a relationship between living in rural areas and acquiring protective environmental factors against the development of asthma and atopy. In our previous study, we found a correlation between particular bacterial species and early-onset wheezing in infants from the rural tropics of Ecuador who were corticosteroid-naïve and had limited antibiotic exposure. We now describe a longitudinal study of infants conducted to determine the age-related changes of the microbiome and its relationship with wheezing.

**Methods:**

We performed an amplicon sequencing of the 16S rRNA bacterial gene from the oropharyngeal samples obtained from 110 infants who had a history of recurrent episodic wheezing sampled at different ages (7, 12, and 24 months) and compared it to the sequencing of the oropharyngeal samples from 150 healthy infants sampled at the same time points. Bioinformatic analyses were conducted using QIIME and R.

**Results:**

As expected, the microbiota diversity consistently increased as the infants grew older. Considering age-based microbiota changes, we found that infants with wheeze had significantly lower species richness than the healthy infants at 7 months, but not at 12 or 24 months. Most of the core and accessory organisms increased in abundance and prevalence with age, except for a few which decreased. At 7 months of age, infants with wheeze had notably higher levels of a single *Streptococcus* operational taxonomic unit and core microbiota member than controls.

**Conclusions:**

In a cohort with limited antibiotic and corticosteroid use, a progressively more complex and diverse respiratory microbial community develops with age. The respiratory microbiota in early life is altered in infants with wheeze, but this does not hold true in older infants.

## Introduction

Asthma is a chronic inflammatory disease of the airways which is characterized by non-specific bronchial hyper-responsiveness and symptoms such as wheezing, dyspnea, and cough (1, 2). Lung function tests are difficult to perform in infants aged <6 years, and their inaccuracy complicates the diagnosis of asthma in this age group (3). Consequently, the presence of recurrent wheezing not related to infections is the most important diagnostic indicator of asthma in this group (4).

The hygiene hypothesis has been used to explain why some populations have a higher frequency of an over-reactive immune system. This hypothesis was initially proposed following epidemiological studies of asthma and atopy (5) and has subsequently been supported by data from studies in both functional immunology and genetics (6). Urbanization, migration, and modernization are critical factors related to asthma risk, which reflect changes in nutrition, exercise, exposure to allergens, antibiotic and vaccine use, microbial exposures, effects of pollution, and psychosocial stressors (7, 8).

The Latin American country Ecuador has regional differences reflected in studies conducted in its rural and urban areas. According to the Global Initiative for Asthma (GINA) criteria, the estimated prevalence of asthma in Ecuador was 8.2% (9). The International Study of Asthma and Allergies in Childhood (ISAAC) studies estimated a 10.9% prevalence of current wheezing in children in the two most populated cities of Quito and Guayaquil (9, 10), which was greater (16.6%) than 0.8% in the rural Pichincha province (7, 11).

Several studies have found that early exposure to infections and antibiotics from infancy is associated with a higher prevalence of asthma and atopy (12, 13). In addition, a relationship between distinct airway microbiome patterns and the development of asthma has been also found. On the one hand, a higher frequency of *Proteobacteria* has been observed in asthmatic adults compared to controls, with *Haemophilus* spp. being more abundant (14). On the other hand, *Bacteroidetes*, *Firmicutes*, and *Actinobacteria* were more common in controls. Bacterial DNA quantification has shown that asthmatics have larger bacterial burdens than controls (15). Culture-based identification of pathogens in a birth cohort showed that *Streptococcus pneumoniae*, *Haemophilus influenzae*, *Moraxella catarrhalis*, and *Staphylococcus aureus* were more common in wheezing children and predicted future asthma development (16). In contrast, a European U-BIOPRED multicenter study found no difference in the comparison between microbiota patterns of asthmatic school-aged children and those with mild/moderate and severe wheezing (17).

In a previous study (18), we used the 16S RNA gene sequencing to discover the differences in the microbiota of oropharyngeal samples collected from infants in rural Ecuador during a wheezing episode and compared the results to those of the non-wheezing controls. Here, we describe a prospective study of the same population in which we investigate the microbiota development in infants with a history of wheezing.

## Materials and methods

### Subjects

A case–control study was performed using samples from infants aged 7, 12, and 24 months. It was designed as part of the ECUAVIDA cohort, which aimed to study the effects of early infant infections on the development of allergic sensitization and allergic diseases. The study was an unselected population-based birth cohort that recruited 2,404 newborns in the rural district of Quinindé in the Esmeraldas Province, Ecuador (19). Detailed data have been collected by maternal questionnaire from the mothers following birth and periodically to 24 months of age. For the present study, a subcohort of the ECUAVIDA was selected based on cohort children's age and clinical characteristics. The Ethics Committees of the Hospital Pedro Vicente Maldonado and Universidad San Francisco de Quito, Ecuador, approved the study protocol. Informed written consent for participation in the study was obtained from parents or legal guardians of children. The study was registered as an observational study (ISRCTN 41239086).

**Figure 1:**
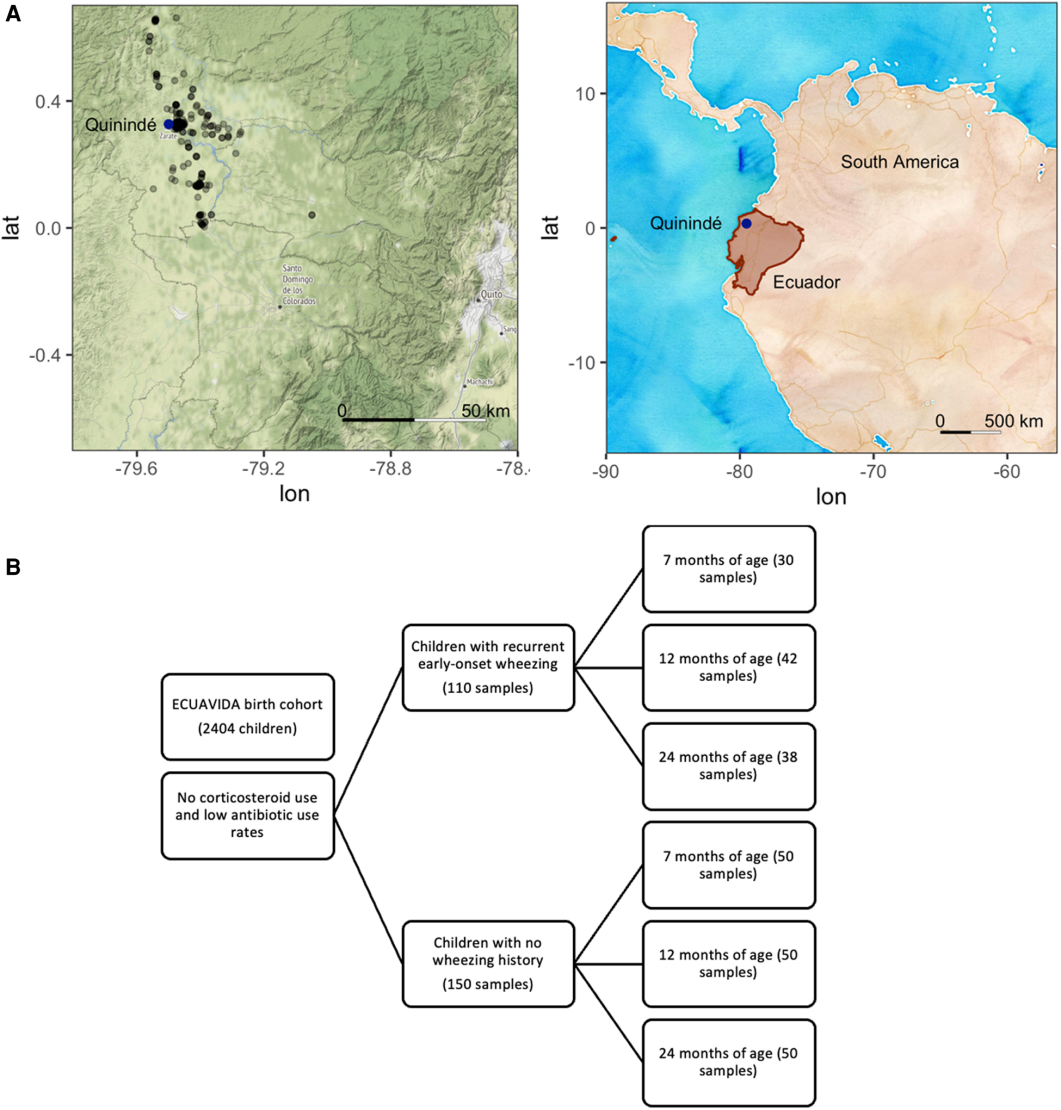
(**A**) Location of Ecuador and Quito and the sampling locations of the ECUAVIDA study in the Quinindé region of rural Ecuador. (**B**) Sample diagram analyzed from the ECUAVIDA cohort.

Oropharyngeal samples were collected from infants with a history of recurrent early-onset wheezing (cases) according to the GINA guidelines (http://www.ginasthma.org/), but not during a wheezing episode. Healthy controls with no wheezing history, current respiratory disease, chronic disease, or current infections were paired by age to cases. Cases and controls were similar regarding ethnicity, area of residence, childhood vaccinations received [Bacillus Calmette–Guerin (BCG), mumps, measles and rubella, tetanus, diphtheria, hepatitis B, and *Haemophilus influenzae* B; Ministry of Public Health of Ecuador (MSP), 2005], and access to essential services (data not shown). There are no differences between groups in delivery type rates, nor does the delivery type account for changes in the microbiota diversity or relative abundance. None had received anti-streptococcal vaccination (which was not compulsory for the Ministry of Public Health of Ecuador then). The location of participants’ households in the Quinindé region of Ecuador is shown in Figure 1A (map tiles by Stamen Design, under CC BY 3.0; data by OpenStreetMap, under ODbL, and by OpenStreetMap, under CC BY SA).

### Sample processing

Samples were collected, transported, and processed with DNA extraction, 16S rRNA gene amplification, and pyrosequencing. Bacterial DNA was extracted from the 400 throat swabs using a modified protocol of the commercial QIAamp DNA Mini Kit (Qiagen) (6). In the final step, 40 µl of nuclease-free water was added instead of the elution buffer supplied by the kit. If the DNA was not used immediately after extraction, samples were stored at −20°C until required.

Polymerase chain reaction (PCR) was used to amplify the variable regions (V3–V5) of the gene that encodes for 16S rRNA in the bacteria. To minimize the PCR nucleotide insertion mistakes, samples were amplified in quadruplicate reactions with 20 cycles each and then pooled. For the PCR, the V3 region of the 16S rRNA gene was amplified using the following conserved primers:

339F (Forward) 5′- CCTACGGGAGGCAGCAG-3′
907R (Reverse) 5′- CCGTCAATTCMTTTRAGT-3′

Each sample was amplified by quadruplication to decrease the PCR nucleotide incorporation errors in further sequencing. PCR reactions and cycling conditions had been previously optimized within the Molecular Genetics and Genomics group at the National Heart and Lung Institute.

The amplicon libraries were quantified using Quant-iT PicoGreen dsDNA Assay Kit according to the manufacturer’s protocols (www.454.com). Subsequently, amplicons were diluted separately to 1 × 10^9^ molecules/µl in 1× tris(hydroxymethyl)aminomethane and ethylenediaminetetraacetic acid (TE) buffer. Then, samples were pooled in a workable solution, with every sample containing the same number of molecules per microliter. During the emulsion PCR prior to pyrosequencing, a maximum of 0.5 molecules of amplicon per bead was calculated to avoid over-enrichment. Nucleic acid extraction, amplification, and sequencing controls were used to determine the possible batch effect bias.

For additional details, please see Supplementary Methods.

### Data analysis

From the raw sequences obtained, data analysis was performed using the third-party microbiological communities’ program QIIME v1.9 (http://qiime.sourceforge.net/) (20). A mapping file was created containing the name of each sample, sample barcode sequence, the linker/primer sequence used to amplify the sample's desired gene, and all the metadata related to each sample: age, gender, case/control, wheezing status, treatment of the subject whom the sample was collected from, and sample type.

Read errors were removed if there were <200 and >800 nucleotides (nt), barcode or primer mismatches, and ambiguous nucleotides and if the quality score was <25. The AmpliconNoise algorithm (21) was used to avoid overestimating the diversity and for chimera removal. Sequences were randomly resampled (rarefied) to 519 reads per sample. A preliminary ordination analysis was carried out to identify four samples that did not cluster with the remaining samples. On inspection of community composition, these outliers were discovered to be dominated by potential respiratory pathogens. The original notes taken during sampling were checked for clinical evidence of current respiratory infections. Two of these individuals had received antibiotics for respiratory infections immediately after sampling; therefore, their samples were removed from the analysis. There was no clinical reason for excluding the other two samples, so they were consequently retained.

Operational taxonomic units (OTUs) were defined using the UCLUST algorithm (22). Sequences were aligned using a Python-based tool employing the Nearest Alignment Space Termination (NAST) algorithm called PyNAST (23) to the Greengenes core reference alignment (24). The alpha diversity coefficients such as Shannon, OTUs observed per sample, evenness, and richness statistics (Chao1) were computed (20)*.* Beta diversity was calculated between groups at the OTU level using Adonis R vegan package (25), and Canberra, Bray–Curtis, UNIFRAC, and weighted UNIFRAC statistics were performed (26). The statistical analysis to compare between groups was performed in R version 4.0.3 (27) using RStudio version 1.4.1103 and phyloseq (28) along with other R vegan packages (24), such as microbiome R package (29), and statistical differences (30), such as Picante (R tools for integrating phylogenies and ecologies), ggplot2 (graphical tool), plyr (tools for splitting, applying, and combining data), Biostrings (string objects representing biological sequences and matching algorithms) (31), ape (analyses of phylogenetics and evolution) (32), and ade4 (analysis of Euclidean data). In generalized least squares modeling, model 1 looked at the relationship between species richness and the age of infants, and model 2 incorporated whether they wheezed or were healthy. Generalized least squares fit by restricted maximum likelihood (REML) are detailed in Supplementary Methods. Statistical analysis of epidemiological data was performed by SPSS version 22.

## Results

Samples from 260 subjects (110 cases and 150 controls) were obtained, and 91 cases and 134 controls remained after sample processing (Figure 1B). Occasionally repeat samples were taken from the same child simultaneously (27 samples). In these cases, the first sample from each child was retained for this analysis to avoid partial repeated measures.

Samples were collected from cases and controls when they had no evidence of a current airway infection (cold symptoms and fever). All infants were corticosteroid-naïve, and none had received antibiotics during the 2 weeks before sampling. The respiratory tract episodes per person were only 0.02 in the case group and 0.04 in the control group. From the clinical records, the use of antibiotics for any reason was 0.06 doses per year per person in the case group and 0.07 in the control group.

### Development of the airways microbiome

**Figure 2:**
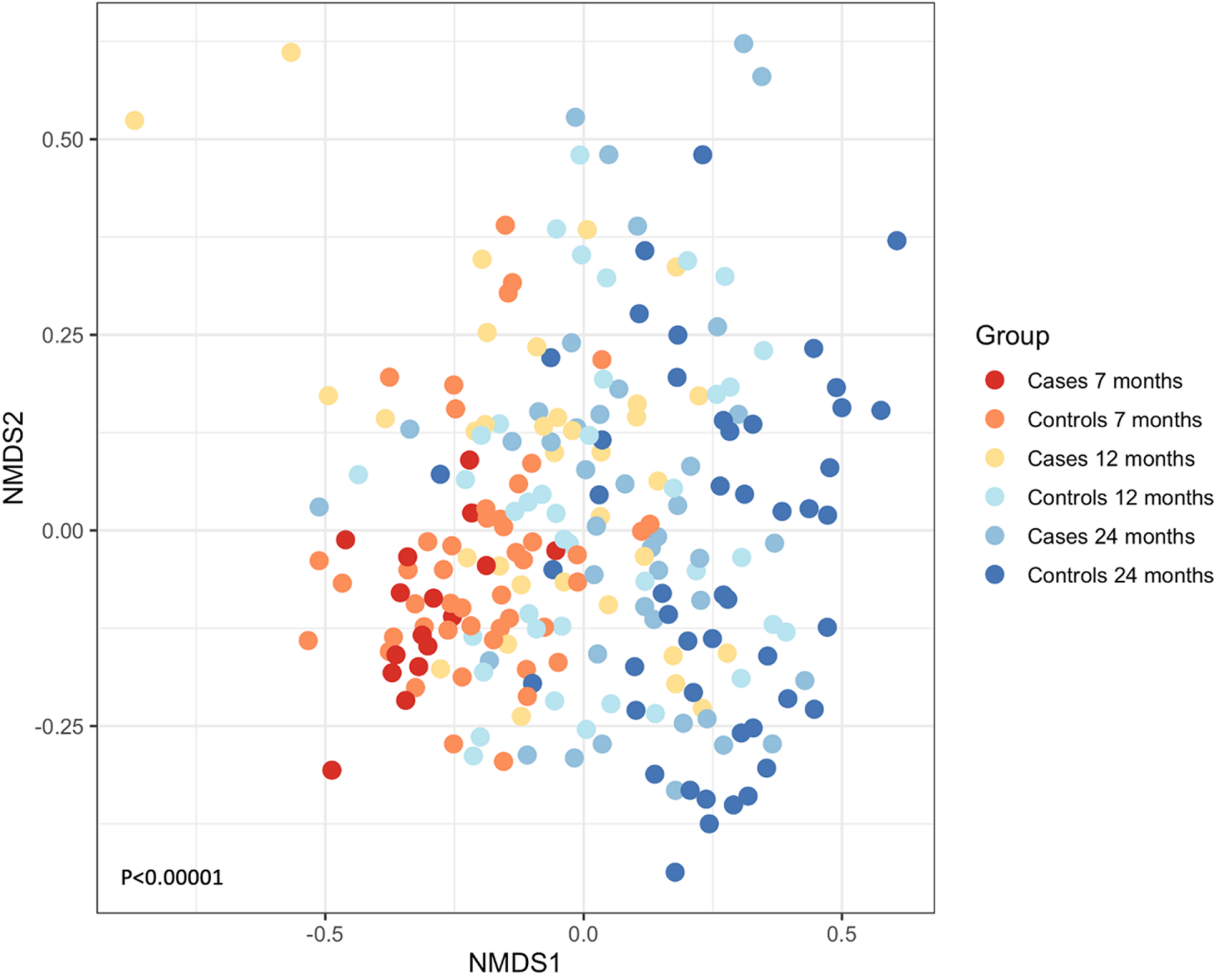
NMDS of the Jaccard similarity between samples, colored by whether they are cases (wheeze) or controls and by age. A shift from lower left to right can be seen for increasing age and health. ANOVA *P* < 0.00001.

A total of 116,775 sequences were analyzed post-filtering, and 796 OTUs were identified from the 225 samples. When cases (*N* = 91) and controls (*N* = 134) were combined, the most abundant OTUs that were present were *Streptococcus* spp., followed by *Neisseria* spp., *Veillonella* spp., *Actinomyces* spp., and *Prevotella* spp. We examined if the composition of the microbial communities changed as the children grew older. Exploratory ordination of the beta diversity of samples, the similarity of communities, and their abundance in each sample using non-metric multidimensional scaling (NMDS) of Jaccard similarity were performed. This was colored by age and whether each sample represented a case or control (Figure 2). The youngest cases appeared at the bottom left and progressed to the oldest controls at the right of the plot, suggesting that microbial community structure might relate to age and wheeze. This was formally tested using the permutational multivariate ANOVA (Adonis). The most significant variation in beta diversity was explained by age, accounting for 9% (*P* < 0.0001). A very small, yet significant, proportion of the diversity was explained by whether samples came from cases or controls (0.9%, *P* = 0.0168).

**Figure 3:**
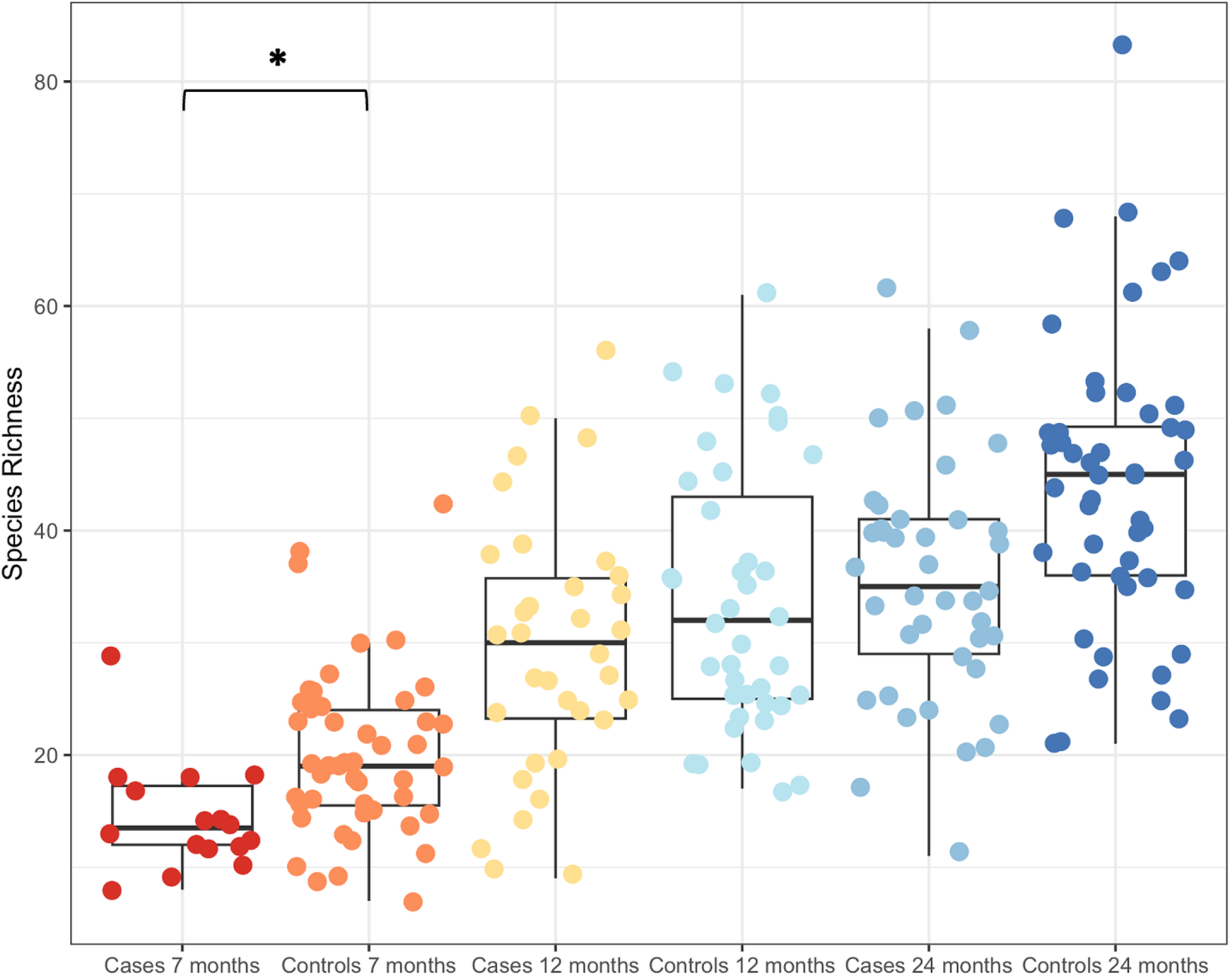
Boxplot of increasing species richness with age. Cases and controls had significantly different species richness at 7 months of age, but not at the two later time points of 12 and 24 months. *P < 0.00001.

Given the small, significant effect of cases and controls in explaining the variance in beta diversity, the effect was explored further. Boxplots of species richness (Figure 3) and other measures of alpha diversity (Shannon's entropy and Simpson's index, not shown) all demonstrated a difference in diversity between cases and controls, particularly at 7 months. Generalized least squares models were constructed to test whether there was an impact of the wheeze variable after considering age. Using this approach, species richness significantly differed with age (*P* < 0.0001). Including wheeze in this model improved the Akaike information criterion (AIC), which was significantly different at 7 months, but not at 12 or 24 months (please see Supplementary Methods for details of the models and model selection).

Other epidemiological variables were compared between cases and controls to determine whether they may have influenced the microbiota patterns or not. Gender, crowdedness of living, maternal education, and parental income were not different between groups (Table 1). Biometric measures at birth, such as weight and height, were not different between both groups and could not explain specific microbiota patterns. The infants included in the study had fewer respiratory tract infections (pharyngitis, pneumonia, and bronchitis). Malnutrition and anemia were present in the clinical records of a few infants in both groups. Still, they were not different between cases and controls, with malnutrition found in 2% of infants in both groups and anemia in 1% and 2% in the case and control groups, respectively. Neither condition was present during sampling, nor it has explained the diversity differences in the diversity analyses.

**Table 1:** Epidemiological characteristics of cases and controls.

	Case	Control	*P *< 0.05
Sex (% male)	54	46	
Number of individuals per room of the house	3.15	3.06	
Average parental income (US$ per month)	222	229	
Average birth weight (g)	3,390	3,341	
Average birth height (cm)	49.6	49.1	
Maternal education (%)			
Illiterate	2%	2%	
Primary incomplete	17%	13%	
Primary complete	23%	22%	
Secondary incomplete	15%	16%	
Secondary complete	32%	44%	*
University incomplete	10%	3%	*
University complete	1%	0%	
Respiratory tract infections (episodes per person)	0.02	0.04	
Antibiotics (numbers of times used per person)	0.06	0.07	
Malnutrition (% reported)	2%	2%	
Anemia (% reported)	1%	3%	

* *P < *0.05. Maternal education refers to the last formal instruction the mother had at the time of the cohort inclusion.

**Figure 4:**
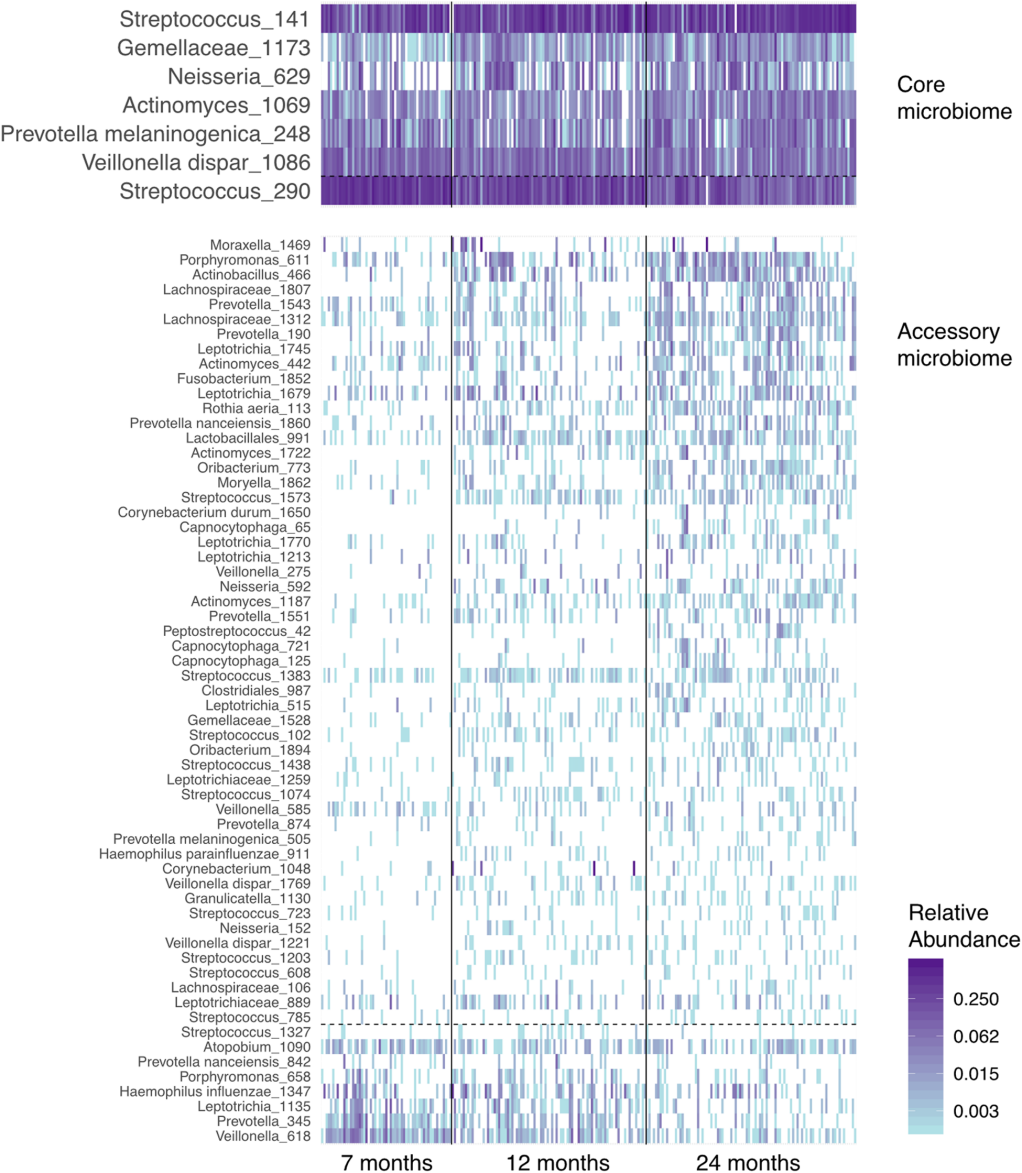
A heatmap of the abundance of the “core” and “accessory” microbiota in airway samples from the ECUAVIDA cohort. Vertical lines divide the age classes of children. Horizontal dashed lines separate OTUs, which increase between 7 and 24 months of age (above the line), and those that decrease (below the line). OTUs in fewer than 10% of samples are not shown, and the “core microbiome” is defined as those in over two-thirds of samples.

Next, we examined the dataset at the level of OTUs, presence, and abundance of individual bacteria (Figure 4). Seven prevalent OTUs formed the “core microbiome” in over two-thirds of all samples. Six of these seven increased in abundance and prevalence with age. The “accessory microbiome” showed a similar trend, with most OTUs accruing and becoming more prevalent with infants’ age. A few decreased with age, potentially representing OTUs replaced by competing strains.

**Figure 5:**
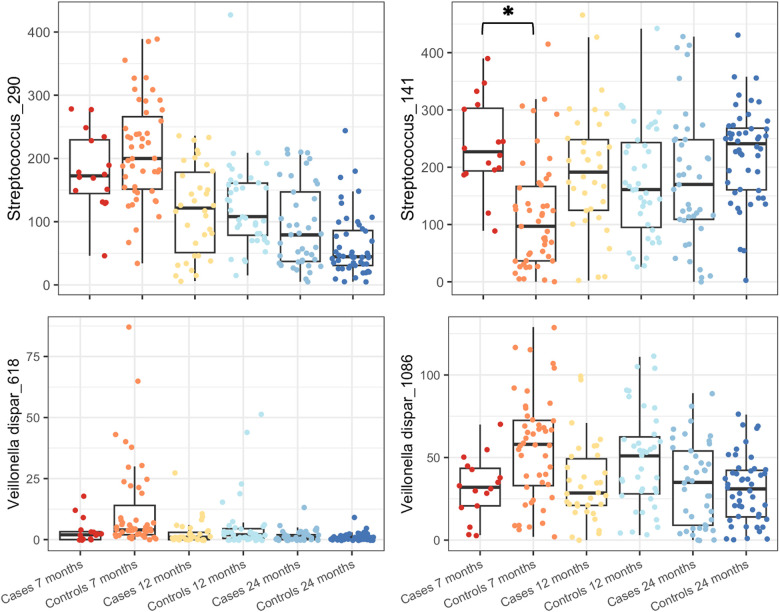
Boxplots of OTU abundances of OTUs identified as significantly different by indicator species analysis and *F*-test are shown for subjects grouped by age and case/control status. *Streptococcus*_141 increased in cases compared to the control group at 7 months of age, and all others were decreased compared to that of the control group at the same age. *P < 0.00001.

Indicator species analysis was used to identify the OTUs associated with cases or controls aged at 7 months. Five OTUs were significantly different at *P* < 0.05 level: *Streptococcus*_141 (*P* = 0.03) was the indicator used in the case group, and *Streptococcus*_290 (*P* = 0.005), *Veillonella dispa*r_618 (*P* = 0.005), *Veillonella dispar*_1086 (*P* = 0.005), and *Prevotella*_345 (*P* = 0.005) were the indicators used in the control group, both aged at 7 months. As indicator species analysis can be sensitive and can pick up differences driven by only a single sample, an *F*-test with Benjamini–Hochberg false discovery rate (FDR) correction was carried out using the mt function of phyloseq. Four of the above OTUs were also significant using this approach (*P*_adj_ = 0.0187, FDR = 0.0078) with *Prevotella*_345 failing to achieve significance (Figure 5).

## Discussion

We did a nested case–control study in infants from the rural tropics of Ecuador, where the prevalence of doctor-diagnosed asthma is low (10). Still, wheezing symptoms occurred at a frequency comparable with urban populations (33). A strength of this study is that the infants had never used inhaled corticosteroids and had low antibiotic usage, allowing investigation of the role of the microbiota in infantile wheezing independently of bias from the consequences of medication. According to the hygiene hypothesis, the rural environment, low antibiotic usage, and high rate of parasitic infections in this population would be protective against the development of asthma and atopic disease (5, 33). The microorganisms’ influence on asthma physiopathology has established that microbiota alterations impact the host's resistance to pathogen colonization (34). Alterations in the normal microbiota may alter the host resistance to pathogen colonization in the gut (35, 36) and in the airways (37, 38) through direct inhibition of pathogen growth by commensal secreted factors (39).

The current study shows that the upper airway microbiota develops with age, principally through acquiring a more complex and diverse microbiota over time (richness indexes in alpha diversity and age clustering in beta diversity indexes). Although the core OTUs remained similar from 7 months until 2 years of age, other minor OTUs appeared through this period. The observed increase in diversity was due to the acquisition of new organisms. The most common bacteria in the infants’ upper airways were *Streptococcus* spp. *Bacteroidales* is also a common phylum in the upper airways, oral and nasopharyngeal microbiota. It includes the genus *Prevotella* which has been considered protective against the development of asthma in adults (14). These results were consistent with previous culture-independent studies of the airway microbiome in European populations (14, 40, 41) but differed from other studies that showed no differences in asthma/wheezing status in oropharyngeal microbiota (17). However, the current study did not cluster the wheezing status in severity compared with U-BIOPRED.

Infants that presented with wheezing at 7 months of age consistently had microbiota patterns that differed from controls paired at the same age. This significant difference was not retained in older age groups, suggesting that wheeze results in an early perturbation that the accrual of OTUs in older children might correct. Indicator species analysis identified one OTU that characterized cases at 7 months of age and three that indicated controls at the same age. All these OTUs were very prevalent, with three core microbiota members present in more than two-thirds of the samples, irrespective of age or wheeze. Significant differences between wheezing and no-wheezing children can also highlight the correlation between the upper airways and gut microbiotas. Several studies have shown that a Mediterranean diet (rich in butyrate) during the first year of life can be related to asthma protection (42, 43), but is not affected by maternal or adult diets (43). However, longitudinal randomized clinical trials are needed to back up this hypothesis. It is also striking that a rural/diverse diet possesses a dose–response effect in atopy and asthma, including food allergies, as shown in the PASTURE study group (44). This can be related to the ECUAVIDA cohort, where a rural, diverse diet can influence the upper airways and gut microbiome (however, we did not record the diet determinants and the effects of diet diversity).

Studies of airway microbiota using culture-independent techniques have not been done previously in Latin American countries where environmental exposure and disease incidence differ significantly from Europe and North America. This project establishes a relationship between wheezing and bacterial microbiota patterns in the airways of children from the rural tropics of Ecuador. During sampling, strict criteria were established to avoid skewed results, for example, caused by current infectious processes, recent use of antibiotics, socioeconomic indexes, or incorrect oropharynx swabbing techniques. The major limitation of the current study was that it was not possible to obtain samples from the same children at all time points, which has not allowed a longitudinal paired analysis. In addition, no records of diet and metabolic determinants were recorded on each subject, which did not allow us to correlate results with gut microbiota. A deeper shotgun metagenomics approach will be considered in a future study to compare ecological features between wheezing and not wheezing infants and an approximation of the metabolic factors affecting the gut microbiota/host relationships.

In conclusion, the upper airway bacterial microbiota differs between infants with wheezing and healthy controls. Although it is not possible to say whether the organisms and diversity differences are in response to a wheezing episode or whether they cause the episode, in this unique cohort, we can demonstrate that this effect is independent of treatment with either corticosteroids or antibiotics and that it affects core members of the oropharyngeal microbiota. Whether these differences are causative or not, they could influence the physiopathology of asthma development later in life and contribute to subsequent chronic inflammation in the airway mucosa.

## Data Availability

The datasets presented in this study can be found in online repositories. The names of the repository/repositories and accession number(s) can be found in the article/Supplementary Material.

## References

[1] Busse WW Lemanske RF. Asthma. N Engl J Med. (2001) 344(5):350–62. 10.1056/nejm200102013440507 11172168

[2] Nelson KA Zorc JJ. Asthma update. Pediatr Clin North Am. (2013) 60(5):1035–48. 10.1016/j.pcl.2013.06.003 24093894

[3] Szefler SJ Chmiel JF Fitzpatrick AM Giacoia G Green TP Jackson DJ Asthma across the ages: knowledge gaps in childhood asthma. J Allergy Clin Immunol. (2014) 133(1):3–14. 10.1016/j.jaci.2013.10.018 24290281 PMC3925634

[4] Wechsler ME. Managing asthma in primary care: putting new guideline recommendations into context. Mayo Clin Proc. (2009) 84(8):707–17. 10.4065/84.8.707 19648388 PMC2719524

[5] Strachan DP. Hay fever, hygiene, and household size. Br Med J. (1989) 299(6710):1259–60. 10.1136/bmj.299.6710.1259 2513902 PMC1838109

[6] Liu AH. Hygiene theory and allergy and asthma prevention. Paediatr Perinat Epidemiol. (2007) 21(S3):2–7. 10.1111/j.1365-3016.2007.00878.x 17935569

[7] Cooper PJ Rodrigues LC Cruz AA Barreto ML. Asthma in Latin America: a public heath challenge and research opportunity. Allergy. (2009) 64(1):5–17. 10.1111/j.1398-9995.2008.01902.x 19076533

[8] Weinberg EG. Urbanization and childhood asthma: an African perspective. J Allergy Clin Immunol. (2000) 105(2):224–31. 10.1016/s0091-6749(00)90069-1 10669840

[9] Masoli M Fabian D Holt S Beasley R, Global Initiative for Asthma (GINA) Program. The global burden of asthma: executive summary of the GINA Dissemination Committee report. Allergy. (2004) 59(5):469–78. 10.1111/j.1398-9995.2004.00526.x 15080825

[10] Mallol J Solé D Baeza-Bacab M Aguirre-Camposano V Soto-Quiros M Baena-Cagnani C Regional variation in asthma symptom prevalence in Latin American children. J Asthma. (2010) 47(6):644–50. 10.3109/02770901003686480 20642377

[11] Weinmayr G Weiland SK Björkstén B Brunekreef B Büchele G Cookson WOC Atopic sensitization and the international variation of asthma symptom prevalence in children. Am J Respir Crit Care Med. (2007) 176(6):565–74. 10.1164/rccm.200607-994OC 17575099

[12] McKeever TM Lewis SA Smith C Collins J Heatlie H Frischer M Early exposure to infections and antibiotics and the incidence of allergic disease: a birth cohort study with the West Midlands general practice research database. J Allergy Clin Immunol. (2002) 109(1):43–50. 10.1067/mai.2002.121016 11799364

[13] Droste JHJ Wieringa MH Weyler JJ Nelen VJ Vermeire PA Van Bever HP. Does the use of antibiotics in early childhood increase the risk of asthma and allergic disease? Clin Exp Allergy. (2000) 30(11):1548–53. 10.1046/j.1365-2222.2000.00939.x 11069562

[14] Hilty M Burke C Pedro H Cardenas P Bush A Bossley C Disordered microbial communities in asthmatic airways. PLoS One. (2010) 5(1):e8578. 10.1371/journal.pone.0008578 20052417 PMC2798952

[15] Huang YJ Nelson CE Brodie EL Desantis TZ Baek MS Liu J Airway microbiota and bronchial hyperresponsiveness in patients with suboptimally controlled asthma. J Allergy Clin Immunol. (2011) 127(2):372–81.e1–3. 10.1016/j.jaci.2010.10.048 21194740 PMC3037020

[16] Bisgaard H Hermansen MN Buchvald F Loland L Halkjaer LB Bønnelykke K Childhood asthma after bacterial colonization of the airway in neonates. N Engl J Med. (2007) 357(15):1487–95. 10.1056/nejmoa052632 17928596

[17] Thorsen J Stokholm J Rasmussen MA Roggenbuck-Wedemeyer M Vissing NH Mortensen MS Asthma and wheeze severity and the oropharyngeal microbiota in children and adolescents. Ann Am Thorac Soc. (2022) 19(12):2031–43. 10.1513/AnnalsATS.202110-1152OC 35904980

[18] Cardenas PA Cooper PJ Cox MJ Chico M Arias C Moffatt MF Upper airways microbiota in antibiotic-naïve wheezing and healthy infants from the tropics of rural Ecuador. PLoS One. (2012) 7(10):e46803. 10.1371/journal.pone.0046803 23071640 PMC3465279

[19] Cooper PJ Chico ME Platts-Mills TA Rodrigues LC Strachan DP Barreto ML. Cohort profile: the Ecuador life (ECUAVIDA) study in Esmeraldas province, Ecuador. Int J Epidemiol. (2015) 44(5):1517–27. 10.1093/ije/dyu128 24990475 PMC4681103

[20] Caporaso JG Kuczynski J Stombaugh J Bittinger K Bushman FD Costello EK QIIME allows analysis of high-throughput community sequencing data. Nat Methods. (2010) 7(5):335–6. 10.1038/nmeth.f.303 20383131 PMC3156573

[21] Reeder J Knight R. Rapidly denoising pyrosequencing amplicon reads by exploiting rank-abundance distributions. Nat Methods. (2010) 7(9):668–9. 10.1038/nmeth0910-668b 20805793 PMC2945879

[22] Edgar RC. Search and clustering orders of magnitude faster than BLAST. Bioinformatics. (2010) 26(19):2460–1. 10.1093/bioinformatics/btq461 20709691

[23] Caporaso JG Bittinger K Bushman FD Desantis TZ Andersen GL Knight R. PyNAST: a flexible tool for aligning sequences to a template alignment. Bioinformatics. (2010) 26(2):266–7. 10.1093/bioinformatics/btp636 19914921 PMC2804299

[24] DeSantis TZ Hugenholtz P Larsen N Rojas M Brodie EL Keller K Greengenes, a chimera-checked 16S rRNA gene database and workbench compatible with ARB. Appl Environ Microbiol. (2006) 72(7):5069–72. 10.1128/AEM.03006-05 16820507 PMC1489311

[25] Dixon P. VEGAN, a package of R functions for community ecology. J Veg Sci. (2003) 14(6):927–30. 10.1111/j.1654-1103.2003.tb02228.x

[26] Lozupone C Lladser ME Knights D Stombaugh J Knight R. UniFrac: an effective distance metric for microbial community comparison. ISME J. (2011) 5(2):169–72. 10.1038/ismej.2010.133 20827291 PMC3105689

[27] R Foundation for Statistical Computing. R: a language and environment for statistical computing. Austria (2020). Available at: http://www.r-project.org/ (Accessed March 2023).

[28] McMurdie PJ Holmes S. phyloseq: an R package for reproducible interactive analysis and graphics of microbiome census data. PLoS One. (2013) 8(4):e61217. 10.1371/journal.pone.0061217 23630581 PMC3632530

[29] Shetty SA Lahti L. Microbiome data science. J Biosci. (2019) 44(5):115. 10.1007/s12038-019-9930-2 31719224

[30] De CM Legendre P. Associations between species and groups of sites: indices and statistical inference. Ecology. (2009) 90(12):3566–74. 10.1890/08-1823.1 20120823

[31] Reimers M Carey VJ. Bioconductor: an open source framework for bioinformatics and computational biology. Methods Enzymol. (2006) 411:119–34. 10.1016/S0076-6879(06)11008-3 16939789

[32] Paradis E Claude J Strimmer K. APE: analyses of phylogenetics and evolution in R language. Bioinformatics. (2004) 20(2):289–90. 10.1093/bioinformatics/btg412 14734327

[33] Cooper PJ Vaca M Rodriguez A Chico ME Santos DN Rodrigues LC Hygiene, atopy and wheeze-eczema-rhinitis symptoms in schoolchildren from urban and rural Ecuador. Thorax. (2014) 69(3):232–9. 10.1136/thoraxjnl-2013-203818 24105783 PMC3932750

[34] Sachs APE Van der Waaij D Groenier KH Koeter GH Schiphuis J. Oropharyngeal flora in asthma and in chronic obstructive pulmonary disease. Indigenous oropharyngeal microorganisms in outpatients with asthma or chronic obstructive pulmonary disease. Am Rev Respir Dis. (1993) 148(5):1302–7. 10.1164/ajrccm/148.5.1302 8239167

[35] Artis D. Epithelial-cell recognition of commensal bacteria and maintenance of immune homeostasis in the gut. Nat Rev Immunol. (2008) 8(6):411–20. 10.1038/nri2316 18469830

[36] Lunjani N Walsh LJ Venter C Power M MacSharry J Murphy DM Environmental influences on childhood asthma-the effect of diet and microbiome on asthma. Pediatr Allergy Immunol. (2022) 33(12):e13892. 10.1111/pai.13892 36564884 PMC10107834

[37] Crowe CC Eugene Sanders W Longley S. Bacterial interference. II. Role of the normal throat flora in prevention of colonization by group A *Streptococcus*. J Infect Dis. (1973) 128(4):427–32. 10.1093/infdis/128.4.527 4147559

[38] Insel M Kraft M. Bacteria in asthma pathogenesis. Immunol Allergy Clin North. (2019) 39(3):377–89. 10.1016/j.iac.2019.03.006 31284927

[39] Tano K Håkansson EG Wallbrandt P Rönnqvist D Holm SE Hellström S. Is hydrogen peroxide responsible for the inhibitory activity of alpha-haemolytic streptococci sampled from the nasopharynx? Acta Otolaryngol. (2003) 123(6):724–9. 10.1080/00016480310000403 12953772

[40] Depner M Ege MJ Cox MJ Dwyer S Walker AW Birzele LT Bacterial microbiota of the upper respiratory tract and childhood asthma. J Allergy Clin Immunol. (2017) 139(3):826–34.e13. 10.1016/j.jaci.2016.05.050 27576124

[41] Ege MJ Mayer M Normand AC Genuneit J Cookson WOCM Braun-Fahrländer C Exposure to environmental microorganisms and childhood asthma. N Engl J Med. (2011) 364(8):701–9. 10.1056/nejmoa1007302 21345099

[42] Papamichael MM Itsiopoulos C Susanto NH Erbas B. Does adherence to the Mediterranean dietary pattern reduce asthma symptoms in children? A systematic review of observational studies. Public Health Nutr. (2017) 20(15):2722–34. 10.1017/S1368980017001823 28803594 PMC10261538

[43] Lv N Xiao L Ma J. Dietary pattern and asthma: a systematic review and meta-analysis. J Asthma Allergy. (2014) 7(7):105–21. 10.2147/JAA.S49960 25143747 PMC4137988

[44] Roduit C Frei R Depner M Schaub B Loss G Genuneit J Increased food diversity in the first year of life is inversely associated with allergic diseases. J Allergy Clin Immunol. (2014) 133(4):1056–64. 10.1016/j.jaci.2013.12.1044 24508301

