# Supplementary material for: Delayed acquisition of airway commensals in antibiotic naïve children and its relationship with wheezing in rural Ecuador
Source: Front Allergy. 2023 Aug 10;4:1214951. doi: 10.3389/falgy.2023.1214951 (PMC10449644; doi:10.3389/falgy.2023.1214951)
Supplement: Supplementary file 1 [file Datasheet1.docx]

Supplementary Material

Delayed acquisition of airway commensals in antibiotic naïve children in rural Ecuador.

Paul A Cardenas, MD, PhD^1,2*^, Michael J Cox, PhD^1†^, Saffron A Willis-Owen^1^, Miriam F Moffatt, DPhil^1^, William O Cookson, DPhil^1^, Phillip J Cooper, PhD^3,4^

^*^**Correspondence:**Paul A. Cardenas
pacardenas@usfq.edu.ec

# Supplementary methods

# Bacterial DNA extraction from Throat Swabs:

# Bacterial DNA was extracted from the 400 throat swabs using a modified protocol of the commercial QIAmp DNA Mini Kit (Qiagen).

# Each swab head was transferred into a 2 ml microcentrifuge tube, and 432µl TE (Tris EDTA pH 8.0) plus 18µl 4X lysozyme solution was added (4X lysozyme in a concentration of 1000U/µl was prepared from lysozyme stock at 30,000 U/µl Ready-Lyse™ Lysozyme Solution of EPICENTRE, UK).

# Samples were incubated for 1 hour at 37°C to allow improved Gram +ve bacterial walls lysis. During this hour, samples were vortexed for 20 seconds at intervals of 15 minutes. Next 30μl of Proteinase K and 450μl of Buffer AL were added to the tube and samples were incubated at 56°C for 30 minutes. To terminate the Proteinase K step, samples were incubated for 5 min at 95°C.

# Added 450μl of Ethanol (96–100%) to the sample and vortexed to obtain a homogeneous solution. This solution was then applied to the QIAamp Spin Column as per the manufacturer’s protocol.

# In the final step 40μl of nuclease-free water was added instead of the elution buffer supplied by the kit. If the DNA was not being used immediately after extraction, samples were stored at -20°C until required.

# Pyrosequencing Analysis of the Microbiome

# Amplification of the 16S rRNA Gene

# Polymerase chain reaction (PCR) was used to amplify the variable region 3 to 5 of the gene that encodes for 16S rRNA in bacteria. To minimize the PCR nucleotide insertion mistakes, samples were amplified in quadruplicate reactions with 20 cycles each and then pooled.

# For the PCR, the V3 region of the 16S rRNA gene was amplified using the following conserved primers:

# 339F (Forward) 5’- CCTACGGGAGGCAGCAG-3'

# 907R (Reverse) 5'- CCGTCAATTCMTTTRAGT-3’

# The Adaptor Sequence in both the reverse and forward primers is 5'- CCATCTCATCCCTGCGTGTCTCCGACTCAG-3’ and is used to link the biotynilated 5’ extreme of the amplicon with a single Enzyme bead in the Emulsion PCR further step described below.

# Multiplexing was realized using the MIDs 1-300 according to US Human Microbiome Project barcode sequences (12 nucleotides).

# Reactions with a final volume of 25μl were set up containing: 1μl of template DNA, 2.5μl 10X buffer (Roche Applied Biosystems, UK), 1.3% DMSO, 1 μM of each primer (μM), 0.5μl dNTPs (10 mM each of A, T, C and G) and 1.25 U FastStart Hifi Polymerase (Roche Applied Biosystems). The following cycling conditions used were:

# Hot start with 92°C for 2 min,

# 30 cycles of 94°C for 20s, 50°C for 30s and 72°C for 5min,

# Final hold 4°C.

# Each sample was amplified by quadruplicated to decrease the PCR nucleotide incorporation errors in the further sequencing. PCR reactions and cycling conditions had previously been optimized within the Molecular Genetics and Genomics Group at the National Heart and Lung Institute.

# Amplicon Cleaning

# After the PCR amplifications, amplicons were purified using the AMPure XP beads (Roche, UK) to eliminate the primer dimer products and the PCR reaction remnants. For this purpose, I have modified and standardized a protocol of DNA cleaning using 96 wells plates instead of tubes. For this purpose, the Magnetic plate Ambion® #AM10027, 96 well round bottom plates Costar® 3367.

# To purify the desired Amplicon removing smaller PCR products and primers, the ratio 1:1 in relation of AMPure beads volume and PCR product was modified for a 0.7:1 ratio.

# At the beginning, the 4 PCR products were pooled and 100 μl transferred into the round bottom plate. The AMPure bead bottle was vortexed for 20 seconds (or until the beads are entirely resuspended), and 70 μl of AMPure beads were transferred to each well, mixing thoroughly by pipetting up and down at least 12 times until the mixture was homogeneous.

# Plates were incubated for 10 min at room temperature and then placed on the 96-well magnetic stand for 5 min until the beads were in the bottom and the supernatant removed. The beads were washed twice with 100 μl of 70% ethanol on each well. Plates were then incubated at room temperature placed in the magnetic stand until all pellets were completely dry (10-20 min). After this time, they were resuspended on 50 μl of 1x TE on each well, and the supernatant with the DNA was transferred into a new plate and stored at -15 to -25ºC until the quantitation step.

# DNA quantification

# DNA was quantified twice during the protocol, firstly after the 16SrRNA PCR and cleaning, and after the samples pooling (library creation). On both, it was used the Quant-iT PicoGreen dsDNA Assay Kit® from Life Technologies™.

# At the beginning, using the provided PicoGreen reagent (100 ng/μl) there were created 8 standard tubes at a decreasing DNA concentration: 100 ng, 50 ng, 25 ng, 12.5 ng, 6.25 ng, 3.13 ng, 1.56 ng and 0.0 ng. Standards were plated by duplicate on the 96-well black fluorometer plates.

# One microliter of each sample was diluted in 99 µl of TE and pipetted on the 96-well black fluorometer plates. Both on the sample’s wells and standards a solution of 1:200 dilution of PicoGreen reagent was added prior measuring. The samples were excited at 480 nm and the fluorescence emission intensity was measured at 520 nm using a spectrofluorometer.

# It was verified each time that the R2 value of the standard curve is at least 0.98. To calculate each sample’s DNA concentration, the value of curve equation obtained was used. From each measure when y = ax + b, the fluorometry value of each sample was subtracted from b and divided to a.

# Pyrosequencing

# After getting the Amplicon libraries they were quantified using Quant-it Picogreen dsDNA Assay Kit according to the manufacturer protocols (www.454.com). The number of molecules per μL was calculated using the following formula:

# Subsequently amplicons were diluted separately to 1x109 molecules/μL in 1x TE Buffer. Then, samples were pooled in a work solution with every sample containing the same number of molecules per μL. During the Emulsion PCR prior pyrosequencing, it was calculated a maximum of 0.5 molecules of amplicon per bead to avoid over-enrichment using the following formula:

# Emulsion PCR was carried on using the pooled amplicons using the Enzyme beads linked to the adaptor sequences of amplicons. To obtain better results during the emulsion amplification the master mix used was modified from the Roche’s manual: Mol. Bio. Grade Water 397.5 µl, Additive 515 µl, Amp Mix 282.5 µl, Amp Primer 80 µl, Enzyme Mix 70 µl, PPiase 2 µl on a total volume of 1347 µl.

# Beads containing the sample DNA were captured using a vacuum pump and cleaned from the emulsion oil using isopropanol, ethanol, and, finally, the enhancing buffer provided on the kit. Only the beads ligated to an amplicon were revered using magnetic filtering. The success of this step was validated by quantifying the number of beads that had DNA ligated; only if the result was around 500,000 beads (using the beads counter provided by Roche), it proceeded with the next steps.

# During sequencing, the 454 technology uses Enzyme Beads (containing sulfurylase and luciferase) placed onto a PicoTiterPlate device to ensure that the DNA remains positioned in the wells during the sequencing reaction. The DNA ligated to the beads was sequenced using the GS Junior Titanium machine of 454 Roche pyrosequencing technologies.

# Supplementary Figures and Tables

# Statistical packages used

# P*icante (R tools for integrating phylogenies and ecologie), ggplot2 (graphical tool), plyr (tools for splitting, applying and combining data), Biostrings (string objects representing biological sequences and matching algorithms) (24), vegan (community ecology package) (25), ape (analyses of phylogenetics and evolution) (26), ade4 (analysis of Euclidean data) (27).*

# *Generalized least squares modelling*

# *Model one looked at the relationship between species richness and age of infant. Model two incorporated whether they had wheeze or were healthy*

# Generalized least squares fit by REML

Model: Rich ~ Age_months_class * Treatment

Data: ec_data

AIC BIC logLik

1679.83 1703.553 -832.9149

Coefficients:

Value Std.Error t-value p-value

(Intercept) 13.250000 2.583588 5.128527 0.0000

Age_months_classTwelve 16.632353 3.133060 5.308660 0.0000

Age_months_classTwenty_four 22.798780 3.046272 7.484157 0.0000

TreatmentControls 6.4734042.991194 2.164154 0.0315

Age_months_classTwelve:TreatmentControls -3.099347 3.850560 -0.804908 0.4217

Age_months_classTwenty_four:TreatmentControls 1.561149 3.711747 0.420597 0.6745

Model selection based on AICc:

|  | K | AICc | ΔAICc | AICcWt | Cum.Wt | Res.LL |
| --- | --- | --- | --- | --- | --- | --- |
| Mod 2 | 7 | 1680.35 | 0 | 1 | 1 | -832.91 |
| Mod 1 | 4 | 1704.43 | 24.08 | 0 | 1 | -848.12 |
